# Voluntary medical male circumcision and sexual practices among sexually active circumcised men in Mzuzu, Malawi: a cross-sectional study

**DOI:** 10.1186/s12889-020-8309-5

**Published:** 2020-02-11

**Authors:** Zimveka Jones Chatsika, Andrew Kumitawa, Vincent Samuel, Steven Chifundo Azizi, Vincent C. Jumbe

**Affiliations:** 1 College of Medicine, Public Health Department, P/Bag 360, Chichiri, Blantyre 3, Malawi; 2 Malawi Defence Force, Malawi Military Health Services, Moyale Barracks, P.O Box 23, Mzuzu, Malawi; 3 College of Medicine, Research Support Centre, P/Bag 360, Chichiri, Blantyre 3, Malawi; 4 Department of Epidemiology and Biostatistics, School of Public Health, University of Zambia, Post Box 50110, Lusaka, Zambia; 5 Malawi Defence Force, Malawi Military Health Services, Kamuzu Barracks, Private Bag 43, Lilongwe, Malawi; 6 College of Medicine, Department of Public health, Health Systems and Policy, P/Bag 360, Chichiri, Blantyre 3, Malawi

**Keywords:** Voluntary medical male circumcision, Risky practice, HIV, Condom, Transactional sex, Malawi

## Abstract

**Background:**

Voluntary Medical Male Circumcision (VMMC) is one of the strategies being promoted to prevent sexual heterosexual transmission of HIV. It has been adopted by 14 countries with high HIV prevalence and low circumcision rates. The 60.0% protective efficacy of VMMC has come with misconceptions in some societies in Malawi, hence VMMC clients may opt for risky sexual practices owing to its perceived protective effect. The study estimated proportion of circumcised men engaging in risky sexual behaviors post-VMMC, assessed knowledge on VMMC protective effect and identified socio-demographic factors associated with risky sexual practices.

**Method:**

A cross sectional study was conducted at two sites of Mzuzu city. Systematic random sampling was used to select 322 participants aged 18–49 who had undergone VMMC. The independent variables included age, location, occupation, religion, marital status and education. Outcome variables were non condom use, having multiple sexual partners and engaging in transactional sex. Data from questionnaires was analyzed using Pearson’s chi square test and logistic regression.

**Results:**

Out of 322 respondents, 84.8% (273) understood the partial protection offered by VMMC in HIV prevention. Ninety-six percent of the participants self-reported continued use of condoms post VMMC. Overall 23.7–38.3% participants self-reported engaging in risky sexual practices post VMMC, 23.7% (76) had more than one sexual partner; 29.2% (94) paid for sex while 39.9% (*n* = 187) did not use a condom. Residing in high density areas was associated with non-condom use, (*p* = 0.043). Being single (*p* < 0.001), and residing in low density areas (*p* = 0.004) was associated with engaging in transactional sex.

**Conclusion:**

Risky sexual practices are evident among participants that have undergone VMMC. Messages on safer sexual practices and limitations of VMMC need to be emphasized to clients, especially unmarried or single and those residing in low density areas.

## Background

HIV and AIDS pose a major threat to health and livelihood world-wide. Of the 37 million people living with HIV worldwide, 70.0% of these live in sub-Saharan Africa and Malawi accounts for 4.0% [1–3]. Malawi has an HIV prevalence of 8.8% among men and women aged 15–49 [2]. Approximately 5600 people worldwide contract HIV daily [1, 4] and 790,000 adults and children died of AIDS related illnesses, accounting for 66.0% of AIDS related deaths worldwide [1, 4]. An estimated 1 million people in the Sub Saharan region contract HIV annually as of 2017 [1, 4, 5]. In 2017, Malawi registered 39,000 new HIV infections and 17,000 deaths due to AIDS related illnesses in all ages [1, 4]. Heterosexual contact is the major mode of HIV transmission and accounts for 88.0% of the infections [2]. Access to prevention and treatment from this incurable disease is limited in sub-Saharan Africa and the disease affects mostly the productive age group [6]. In this regard, efforts tailored at reducing HIV transmission and averting deaths is a welcome development. However, there are several types of prevention strategies to reduce the risk of acquiring or transmitting HIV such as behavioral, biomedical and structural that are complementary [7, 8].

Voluntary medical male circumcision (VMMC) is a biological intervention and an effective strategy in reducing sexual transmission of HIV from women to men [9–11]. In 2007, the Joint United Nations Programme on HIV/AIDS (UNAIDS) and the World Health Organization (WHO) recommended scale up of Male Circumcision (MC) and has been adopted as one of the HIV preventive strategies in countries with low levels of MC [12]. Globally, the estimated prevalence rate of male circumcision is around 38.7% with half of the circumcisions conducted for religious and cultural reasons [13, 14]. Only 28.0% of the men aged 15–49 in Malawi are circumcised [3]. This figure includes 18.0% circumcised by traditional practitioners and 9.0% by medical professionals [3]. Hence the percentage for those that undergo VMMC is significantly low. Male circumcision is effective when done between the ages of 15 to 20 years and its effect declines with increase in age as the risk of contracting HIV decreases [15].

Evidence of male circumcision on HIV prevention came from meta-analysis of three randomized controlled trials involving 11,050 men that were conducted in South Africa, Kenya, and Uganda which showed a relative risk reduction for contracting HIV infection in circumcised men to be around 60.0% (95% CI 40.0–67.0%) [16, 17]. Consequently, recommendations were made by WHO and UNAIDS to countries with low rates of male circumcision and high HIV prevalence rate to adopt VMMC as a measure to prevent HIV transmission alongside other preventive strategies such as condom use. Following this development, Malawi as one of 14 prioritized countries according to WHO and UNAIDS, adopted and rolled out VMMC in 2011 as one of its HIV preventive strategies [18, 19]. A total of 266,176 VMMCs have so far been performed as of 2015 representing 13.0% of the target that was set in 2011 [20]. Circumcising more than 80.0% of males aged 15 to 49 in a high HIV prevalent region could avoid 3.36 million infections and 386,000 deaths by 2025 [21].

VMMC clients undergo counselling and HIV testing prior to the circumcision procedure where they are informed about the advantages of VMMC and the need to use other HIV prevention strategies including being faithful to one partner, abstaining from sex for a period of 6 weeks following VMMC, and also consistent and correct condom use [22, 23]. During counselling, the protective effect of VMMC which is at 60.0% (as a scientific explanation) is interpreted as a lowered risk of contracting HIV if circumcised [22, 23]. This explanation creates and leaves room for misconceptions and poses a considerable threat to the main preventive effort [23]. It was also noted that the media utilizes this ‘60.0%’ protection in promoting VMMC messages against HIV infection, which was observed in a trial by Auvert et al. [9].

A study conducted in Kenya involving 1344 men showed that 30.7% were involved in early resumption of sex [24]. This is one of the risky sexual practices following VMMC, which may be as a result of the misunderstanding of the protective effect of VMMC [24]. Furthermore, the desire to be circumcised was associated with knowledge of the perceived protective effect of VMMC among the uncircumcised individuals who were practicing risky sexual behaviours as well as high incidence of STIs in circumcised clients [21, 25]. In this case, the motivation to get circumcised could be associated with the desire to freely engage in risky sexual practices due to the perceived protective effect of VMMC. This may point to the lack of understanding as regards the protective effect of VMMC, a development which may contribute to further risky sexual practice among circumcised men hence putting them at risk of contracting HIV.

It is worth noting that womens’ desire for circumcised men, enhanced sexual pleasure, religion, proven safety, affordability, confidentiality and being hygienic prompted participants to go for circumcision [19, 26]. In Malawi, the desire to have more women was the motivating factor for the adolescent boys to get circumcised [21]. There are limited studies on risky sexual practices associated with “60.0% protective effect” of VMMC in Malawi [27]. This study was conducted to determine if circumcised men were likely to engage in risky sexual practices in Mzuzu City owing to the perceived protective effect that VMMC offers.

## Methods

### Study design and study setting

A cross section study was conducted in two health facilities of Mzuzu city located in the northern region of Malawi between 12 February, 2017 and 11 May, 2017. Mzuzu city was chosen since it has two public health facilities that provide free VMMC services to the community with support from partners Jhpiego Malawi and Project Concern International (PCI) Malawi.

### Study population

All men aged 18 to 49 years who underwent voluntary medical male circumcision were eligible for the study.

### Inclusion criteria and exclusion criteria

The study included men aged 18 to 49 years that had undergone VMMC at any of the two sites and 6 months had elapsed following procedure were eligible for the study. On the contrary, those who were very sick were not included in the study.

### Variables

The explanatory variables included socio demographic characteristics; age, occupation, marital status, education, religion and place of residence. Outcome variables were risky sexual practices. Risky sexual practice constituted three indicators namely: transactional sex in all men who reported to have ever engaged in transactional sex, multiple sexual partners in those that had more than one sexual partner and non-condom use in those that did not use a condom during last sex to a non-marital or cohabiting partner.

### Sampling and sample size determination

The two government owned facilities that provide free VMMC services were both used to sample participants for the study. Sample size was determined using STATA version 12 with 31.0% proportion [24], level of statistical significance at 5.0, 95% confidence interval and margin of error; 0.05. The calculated overall sample size was 322 participants.

Systematic random sampling was used to select study participants. Those waiting to collect medication at the pharmacy following consultation were approached. Every 5th male client was selected until the sample size was reached.

### Data collection

An interviewer administered structured questionnaire [28] was used to collect data. Data was collected by two Research Assistants (RAs) and the Principle Investigator (PI). Research assistants were trained in data collection methods and management for 3 days, followed by 1 day of fieldwork pre-testing. The questionnaire had three components: socio-demographic factors, sexual practice assessment and the interpretation of VMMC messaging to prevent HIV. The English questionnaire was translated into *Chitumbuka* and *Chichewa* languages. The translated questionnaires were then back-translated to English to maintain meaning and consistency. The tool was piloted with 10 participants and was amended to capture all the relevant information.

The PI and the RAs approached potential research participants and sought their informed consent prior to data collection. At the end of each day, the PI checked the questionnaires for completeness and consistency and provided feedback to data collectors where necessary. Data was double entered into a secure password protected Microsoft Excel (2007) database and cleaned before analysis. Statistical analysis was done using STATA version 12 (Stata corp., college station, Texas: Statacorp LP, USA).

### Data analysis

Proportions and frequencies for categorical variables were calculated. Pearson’s Chi-square test was used to analyze categorical data for associations and relationships between the variables. Odds ratio was used as a measure of effect. Logistic regression was also used to control for confounding. Statistical tests were assessed at significance level of 0.05 and confidence level of 95%.

### Ethical approval and consent to participate

Ethical approval was obtained from the College of Medicine Research Ethics Committee in Malawi (reference number P.10/16/2035). Written permission to conduct the study was obtained from Mzuzu and Moyale health facilities. Informed written consent were obtained from study participants. Willingness to participate in the study was confirmed by signing or thumb printing on the informed consent sheet.

## Results

### Socio-demographic characteristics of the study participants

Out of the 322 participants, 154 (47.8%) were recruited from Mzuzu Health Centre and 168 (52.2%) were from Moyale Health Facility. The participants’ mean age was 27.6 years SD ± 6.8 (range: 18 to 48). Two-thirds of the sample comprised of men aged 18 to 29, representing 66.8% of total sample; 51.2% of the participants were married, 66.8% came from high density locations, 72.4% attained secondary education, 83.6% were Christians and 34.8% were students (Table 1).

**Table 1:** Socio-demographic characteristics of participants (*n* = 322)

Characteristic	n	%
Age		
18–24	131	40.7
25–29	84	26.1
30–34	55	17.1
35–39	30	9.3
40–49	22	6.8
Marital status		
Single	153	47.5
Married	165	51.2
Widowed	1	0.3
Divorced/Separated	3	0.9
Area of residence		
Low density	107	33.2
High density	215	66.8
Education level		
Primary	48	14.9
Secondary	233	72.6
Tertiary	41	12.7
Religion		
Christian	269	83.5
Islam	38	11.8
Other	15	4.7
Employment status		
Not employed	35	10.9
Student	112	34.8
Business	90	28.0
Farmer	26	8.1
Formal employment	59	18.3

### Proportion of participants engaging in risky sexual practices

Sexual practices of study participants post VMMC showed that the proportion of participants that self-reported engaging in risky sexual practice post VMMC was 38.2% (*n* = 322). About 23.7% (*n* = 322) reported having multiple sexual partners; 29.2% (*n* = 322) reported being involved in transactional sex while 36.9% (*n* = 187) reported non-condom use in the past 6 months. On the other hand, 32.3% (104) had sex prior to the recommended 6 weeks abstinence period (VMMC healing period) (Table 2).

**Table 2:** Proportions of participants that engaged in risky sexual practice

Variable	Number	%	95% CI
No condom use	69 (*n* = 187)	36.9	29.91, 43.87
Paid sex	94 (*n* = 322)	29.2	23.90, 33.85
Multiple sex partners	76 (*n* = 322)	23.6	18.94, 28.27
Overall proportion	123 (*n* = 322)	38.2	32.86, 43.53

### Perceived protective effect of VMMC

The results showed that 50.0% (n = 322) of the participants self-reported undergoing VMMC for HIV prevention, 30.4% for hygiene purposes and 19.6% for other reasons (tradition 2.2%, religion 5.9%, medical reasons 5.3%, and peer pressure 6.2%). The results also show that 84.8% of the respondents reported understanding the partial protection offered by VMMC and that one can still acquire the virus even after circumcision. Only 12.7% of the participants reported that VMMC provides total protection to HIV infection and 2.5% were unsure. On the benefits offered by VMMC, 58.1% stated VMMC helps in the prevention of HIV and STIs, while 37.3% sited hygienic reasons and 4.7% felt it was for sexual pleasure. When participants were asked about the fear of contracting HIV following VMMC, 45.7% of participants stated that they have no fear of contracting HIV following VMMC while 54.4% are still afraid of contracting HIV despite having undergone VMMC. On condom use post VMMC, 94.4% agreed to continued condom use following circumcision. In addition, participants understand that a circumcised man can contract HIV (96.5%) and that an infected circumcised man can still transmit HIV (98.4%) (Table 3).

**Table 3:** Perception of the protective effect offered by VMMC (*n* = 322)

Reasons for undergoing VMMC	n	%
HIV prevention	161	50.0
Hygiene	98	30.4
Others	73	19.6
Perceived understanding of VMMC protection against HIV transmission (*n* = 322)		
Item	n	%
Provides total protection	41	12.7
Provides partial protection	273	84.8
Does not understand	8	2.5
Benefits of VMMC (*n* = 322)	n	%
Prevention of HIV, STI & cancer	187	58.1
Hygiene	120	37.3
Sexual pleasure	15	4.7
VMMC and fears of HIV (*n* = 322)	n	%
Fear reduced	147	45.7
Fear not reduced	175	54.4
Condom use following VMMC	n	%
Continued use	304	94.4
No condom use	18	5.6
Transmission of HIV in the infected circumcised	n	%
Can still transmit HIV	317	98.5
Cannot transmit HIV	5	1.6
Contracting HIV following VMMC	n	%
Can contract HIV	311	96.6
Cannot contract HIV	11	3.4

### Sexual practices

On the type of sexual partner, it was observed that 43.5% had sex with a marital partner, 29.2% paid for sex while 27.3% had a non-cohabiting partner. Results of the participants’ sexual practices post VMMC showed that 76.4% (*n* = 322) of the participants reported having only one sexual partner. Aside those that did not use a condom because they are married, 22.0% (*n* = 70) of the participants did not use a condom during their last sexual encounter with a non-cohabiting partner. Several reasons were cited for the non-condom use (condoms not available 6.2%, feeling safe due to VMMC 7.1 and 8.7% forgot to use a condom). Of those that had a risky sexual act despite being single or married, 36.9% (*n* = 187) did not use a condom. In addition, 32.3% were involved in sex prior to the recommended 6 weeks abstinence period post VMMC while 11.8% had a sexually transmitted infection (Table 4).

**Table 4:** Characteristics of sexual practices among circumcised participants

Number of sexual partners (*n* = 322)	n	%
One partner	246	76.4
More than one partner	76	23.6
Condom (*n* = 322) use last encounter	n	%
Used condom	118	36.7
Did not use condom	204	63.4
Reason condom not used (*n* = 204)	n	%
Marital partner	134	65.7
Condoms not available	20	9.8
Perceived VMMC protection	22	10.8
Forgot	28	13.7
Type of sexual partner (*n* = 322)	n	%
Marital partner	140	43.4
Paid sex partner	94	29.2
Non cohabiting partner	88	27.3
Abstinence post VMMC (*n* = 322)	n	%
Less than six weeks	104	32.3
More than six weeks	218	67.7
STI (*n* = 322)	n	%
Had STI	38	11.8
No STI	284	88.2

### Socio-demographic factors associated with risky sexual practices

As explained in the data analysis section, non-condom use, multiple sexual partners and being involved in transactional sex are the proxy indicators for risky sexual practices. While several demographic factors were tested for association with the risky sexual practices, the results showed that VMMC participants living in high density areas were less likely to use condoms when having sex with non-cohabiting partners (*p*-value = 0.030). Furthermore, the study showed that those aged between 18 and 29 years, residing in low density locations, being unmarried / single, having secondary and tertiary education, being student and unemployed were significantly associated with being involved in transactional sex (Tablse 5–7).

### Univariate and multivariate analysis on sexual practices

The odds of not using a condom during the last sexual encounter among those from high density residential areas was twice as higher compared to those from low density residential areas (OR = 2.0, 95% CI = 1.05–3.81). Age, marital status, occupation, religion and education were not statistically significant with non-condom use (Table 5).

**Table 5:** Univariate and multivariate analysis on non-condom

Univariate and multivariate logistic regression on non-condom use							
Variable		OR^‡^	*p* - value	95% CI	aOR^†^	*p* -value	95% CI
Age	18–29	1					
	30–49	0.53	0.132	0.23–1.21	0.48	0.244	0.14–1.65
Marital status	Single	1					
	Married	0.71	0.357	0.35–1.46	0.61	0.397	0.19–1.92
Education level	Primary	1					
	Secondary	1.12	0.843	0.36–3.46	1.59	0.473	0.44–5.59
	Tertiary	1.53	0.520	0.42–5.58	3.41	0.112	0.75–15.50
Religion	Christianity	1					
	Islam	1.39	0.563	0.46–4.18	1.87	0.337	0.51–6.75
	Others	7.49	0.077	0.81–67.63	6.81	0.100	0.69–66.87
Area of residence	Low density	1					
	High density	2.00	0.034	1.05–3.81	1.93	0.043	1.02–3.84
Occupation	Formal employment	1					
	Student	1.81	0.279	0.62–5.34	1.18	0.798	0.32–4.48
	Farmer	2.40	0.341	0.40–40.55	1.76	0.599	0.21–14.30
	Business	3.20	0.064	0.92–10.98	3.60	0.083	0.84–15.35
	Unemployed	1.60	0.484	0.43–5.95	1.21	0.808	0.26–5.63

^†^adjusted Odds Ratio, ^‡^Odds Ratio

On multiple sexual partners, the age group 25–29 was significantly associated with having multiple sex partners. The odds of having multiple sexual partners among those aged 25–29 was 2.22 times higher compared to those aged 18–24 (OR = 2.22, 95% CI = 1.18–4.20). The other age groups were not significant (Table 6).

**Table 6:** Univariate and multivariate analysis on multiple sex partners

Univariate and multivariate logistic regression on multiple sexual partners							
Variable		OR^‡^	*p* - value	95% CI	aOR^†^	*p* -value	95% CI
Age	18–24	1					
	25–29	2.22	0.013	1.18–4.20	2.28	0.037	1.05–4.95
	30–34	1.11	0.79	0.41–2.46	1.10	0.859	0.39–3.11
	35–49	0.99	0.988	0.31–3.19	0.95	0.942	0.23–3.85
	40–49	1.91	1.570	0.78–4.67	1.60	0.440	0.48–5.29
Marital status	Single	1					
	Married	1.33	2.81	0.79–2.24	1.04	0.922	0.44–2.49
Education level	Primary	1					
	Secondary	1.62	0.247	0.72–3.66	2.19	0.093	0.88–5.48
	Tertiary	1.83	0.247	0.66–5.11	2.40	0.143	0.74–7.73
Religion	Christianity	1					
	Islam	2.03	0.053	0.99–4.17	2.10	0.066	0.95–4.64
	Others	0.54	0.420	0.12–2.44	0.47	0.343	0.10–2.26
Area of residence	Low density	1					
	High density	1.19	0.530	0.69–2.03	1.06	0.855	0.57–1.10
Occupation	Formal employment	1					
	Student	0.80	0.554	0.38–1.68	0.92	0.883	0.34–2.50
	Farmer	0.88	0.817	0.30–2.60	1.05	0.879	0.33–3.72
	Business	1.13	0.751	0.54–2.37	1.11	0.820	0.46–2.66
	Unemployed	0.61	0.354	0.21–1.75	0.61	0.416	0.43–2.49

^†^adjusted Odds Ratio, ^‡^Odds Ratio

On transactional sex, the odds of being involved in transactional sex among those aged 30–49 was 81.0% less compared to those aged 18–29 (OR = 0.19, 95% CI = 0.09–0.37). The odds of being involved in transaction sex among the married ones was 88.0% less compared to those that were single (OR = 0.12, 95% CI = 0.07–0.22). The odds of being involved in transaction sex among those that reside in high density areas was 55.0% less compared to those that reside in low density areas (OR = 0.45, 95% CI = 0.27–0.74). On occupation; students and those that were unemployed were significantly associated with being involved in transaction sex. The odds of being involved in transaction sex was four times higher among the students compared to those with formal employment (OR = 4.02, 95% CI = 1.80–8.97) and the odds of being involved in transaction sex among those unemployed was six times higher compared to those with formal employment (OR = 5.88, 95% CI = 2.23–15.5) (Table 7).

**Table 7:** Univariate and multivariate analysis on transaction sex

Univariate and multivariate logistic regression on transaction sex							
Variable		OR^‡^	*p -* value	95% CI	aOR^†^	*p* -value	95% CI
Age	18–29	1					
	30–49	0.19	< 0.001	0.09–0.37	0.46	0.118	0.17–1.22
Marital status	Single	1					
	Married	0.12	< 0.001	0.07–0.22	0.19	< 0.001	0.08–0.47
Education level	Primary	1					
	Secondary	4.00.	0.005	1.52–10.52	2.30	0.136	0.77–6.91
	Tertiary	4.46	0.009	1.44–13.79	2.67	0.148	0.71–10.02
Religion	Christianity	1					
	Islam	0.70	0.368	0.32–1.53	1.37	0.516	0.53–3.59
	Others	0.16	0.079	0.02–1.24	0.18	0.127	0.02–1.63
Area of residence	Low density	1					
	High density	0.45	0.002	0.27–0.74	0.41	0.004	0.22–0.76
Occupation	Formal employment	1					
	Student	4.02	0.001	1.80–8.97	0.98	0.977	0.33–2.90
	Farmer	1.01	0.988	0.28–3.63	2.04	0.342	0.47–8.90
	Business	1.11	0.819	0.45–2.73	1.24	0.692	0.42–3.76
	Unemployed	5.88	< 0.001	2.23–15.53	2.97	0.083	0.86–10.15

^†^adjusted Odds Ratio, ^‡^Odds Ratio

### Multivariate analysis

The final multivariate model included location, age, marital status, occupation and education. Of these, those residing in high density location were significantly associated with non-condom use (aOR = 1.98, 95% CI = 1.02–3.84) while being single (aOR = 0.19, 95% CI = 0.08–0.47), and residing in low density location (aOR = 0.41, 95% CI = 0.22–0.76) was significantly associated with being involved in transaction sex (Tables 5, 6 and 7).

## Discussion

Overall, the study found that more than one-third (38.2%) of participants; that had undergone VMMC engaged in risky sexual practices. Only area of residence and marital status seem to play a significant role in the adoption of risky sexual practices among participants that had undergone VMMC in this study.

Residing in high density locations was significantly associated with non-condom use. High density locations of Mzuzu are mostly associated with low socioeconomic status hence access and affordability of condoms may be affected [29]. Low education levels may as well affect condom uptake and usage in those residing in high density locations [3]. This result is similar with findings that were observed in the Malawi Demographic and Health Studies (MDHS) where those residing in high density locations were less likely to use condoms [3]. In this regard, knowledge of the perceived protective effect of VMMC in such circumstances could overshadow the need to use other preventive measures like condom use, hence an increase in risky sexual practices.

Furthermore, being single / unmarried and residing in low density residential areas was significantly associated with being involved in transactional sex. Those residing in low density areas in Mzuzu are associated with wealth and higher education hence the likelihood of affording to pay for sex [3]. Most of the unmarried / singles in low density urban locations are mostly either students or employed and have a source of income. Access to information among those residing in low density location could have had an influenced the better understanding of the benefits that VMMC carries and this could have been the driving force to the risky sexual practice among the single / unmarried. This was also observed in a study done in Uganda where risky sexual behaviors were evident among the singles willing to be circumcised and residing in the urban region [30]. A study conducted in Sub-Saharan countries also demonstrated that the unmarried / singles, engage more in transactional sex compared to their married counterparts [31]. The study also observed that economic status has an influence on transactional sex and residing in low density areas of Mzuzu is associated with wealth and higher levels of education [3, 31].

While anecdotal observations show that there is a better perceived understanding that VMMC protects against contracting HIV, the results in this study show a variation in VMMC client’s views. The study shows that a higher proportion of participants reported understanding that VMMC offers partial protection and that one can still acquire HIV if engages in unsafe sexual practices.

A study conducted done in South Africa showed that 75.5% of participants understood the partial protection offered by VMMC a percentage slightly lower as compared to this study [32] while another study conducted in Botswana, Namibia and Swaziland showed that 9.0–15.0% of the participants believe that circumcised men are fully protected against HIV compared to 12.7% from this current study [33]. Almost all of the participants reported the need to continue using condoms post VMMC and that one can still transmit the virus despite having undergone VMMC. Further to this, more than half of the participants reported being afraid of acquiring HIV if they subject themselves to risky sexual practices despite having undergone VMMC.

The participants’ better understanding of VMMC messages as seen from this study could be attributed to an enhanced IEC strategy by the Ministry of Health (MoH) through the use of mass media as a measure to combat rising pressure of possible risk compensation that is being imagined with the rolling out of VMMC in Malawi. This is further influenced by the availability of wide range of mass media and residing in an urban location [3] where access to information is high among the literate population who have undergone counselling prior to undergoing VMMC.

Nevertheless, 12.7% of the study participants had a perception that VMMC provides total protection against HIV acquisition and 2.5% of the study participants were not certain as to what VMMC offers in HIV prevention. These contrasting views could be attributed to message dissemination in relation to HIV and the protection that VMMC offers. VMMC being a new strategy in HIV prevention, most of the messages are focused on the HIV protection with little emphasis on the limitation of VMMC. Furthermore, to increase the uptake of VMMC in Malawi, campaigns are on the increase and message dissemination does not mostly emphasize on the limitation of VMMC [34]. In addition, participants’ understanding and level of education could also have an impact on the uptake and interpretation of the messages that are packaged for VMMC awareness. In this regard, VMMC is being taken as a preventive measure against contracting HIV with little emphasis on its limitation putting at risk clients who may opt for VMMC and engage in risky sexual practices post VMMC owing to its protective effect.

Close to half (45.7%) of the participants in this study agreed to have less fear of contracting HIV following VMMC, which may explain the discrepancy between knowledge and practice of participants that have undergone VMMC in this study where 38.2% still engaged in risky sexual practice despite knowing the level of protection that VMMC offers.

Contextually, VMMC seems to have opened an opportunity for participants that were previously afraid of engaging in risky sexual acts. A study by Kibira et al demonstrated that willingness to be circumcised was associated with risky sexual practice [30] putting forward an idea that being circumcised is likely to encourage clients to engage in risky sexual practices. Apart from the protection that VMMC offers in preventing HIV transmission, the perceived benefits of VMMC were also observed to be a driving factor for participants to go for VMMC [35].

This study found that 38.2% of the participants were involved in risky sexual practices post VMMC. Having multiple sexual partners constituted 23.7%, being involved in transactional sex 29.2% and non-condom use at 36.9% (*n* = 187). Studies by Kibira et al, Mapoma and Bwalya also demonstrated higher proportions of circumcised participants that were involved in risky sexual practices compared to their uncircumcised counterparts [36, 37]. These findings show that there is an increase in risky sexual practices among circumcised participants. Furthermore, in a comparative study, risky sexual practices were less observed in the uncircumcised participants who showed interest to be circumcised than in the circumcised ones [38]. Similarly, a study done in Uganda observed that risky sexual behaviors were associated with willingness to be circumcised and this was evident among the youth, educated and residing in the urban region [30].

These finding supports the idea that undergoing VMMC is likely to encourage participants to engage in risky sexual acts. Results from a study done in Zimbabwe showed a strong association between willingness to be circumcised in participants that had risky sexual practices (multiple partners, being involved in transactional sex and non-condom use) [25]. This observation may explain the continued risky sexual practices in circumcised participants as being carried forward following circumcision and likely due to the perceived protective effect.

Nonetheless, the desire to have multiple sexual partners and engage in transactional sex influenced men to go for VMMC owing to the perceived protective effect of VMMC as observed from studies that were conducted in Zimbabwe and Botswana [30]. Further to this, a study done in Uganda showed that the HIV prevalence among the circumcised was low even with risky sexual practices [37]. These findings may negatively affect the promotion of safer sex practices among the circumcised and could be a driving factor for clients to opt for VMMC and consequently engage in risky sexual practices. Studies done in Zimbabwe, Zambia, two from Uganda and from the 14 prioritized VMMC countries showed that VMMC participants are engaging in risky sexual practices despite the proportions not being significant [37].

In addition, abstinence post VMMC also impacts on HIV transmission. In this study 32.3% (104) resumed sexual activity before 6 weeks and 63.5% were married. Engaging in sex with their marital partner among the married diffused their fear of contracting HIV. The risk of contracting HIV is higher in those that resume sexual activity in less than 6 weeks [39, 40].

Risky sexual practices are evident in the circumcised participants. Varied estimates have been observed from different studies on the risky sexual practices specifically on non-condom use, having multiple sexual partners and being involved in transactional sex. Proportions of those that self-reported engaging in risky sexual practices from this study (38.2%), support the possibility of risky sexual practices arising from being circumcised. It has also been observed that the driving force for men to undergo VMMC was their risky sexual practice prior to getting circumcised. In addition, almost close to half of the participants self-reported less fear of contracting HIV post VMMC. The findings of this study have shown that VMMC participants are likely to engage in risky sexual practices owing to the perceived protective effect that VMMC offers.

Nevertheless, there are some limitations to this study. This study is cross-sectional and causal inferences cannot be drawn. It is also worth noting that the findings could also be limited by social desirability bias in participants’ self-reporting of sexual risk practices during face to face interviews and also recall bias when reporting on their sexual practices.

## Conclusion

HIV still poses a great threat to the livelihood worldwide with Sub-Saharan African bearing the highest burden of the pandemic. The study found that circumcised men are likely to engage in risky sexual practices owing to the perceived protective effect that VMMC offers. A higher proportion of men that underwent VMMC understand the protection that VMMC offers in preventing HIV transmission and the need to use condoms to avoid contracting the virus. Nevertheless, risky sexual practices were observed among circumcised men. There is need to strengthen the information, education and communication (IEC) component before, during and after circumcision with emphasis on the unmarried / single and those residing in low and high densities as they are at risk of engaging in risky sexual practices post VMMC. However, misunderstandings and inaccurate perceptions about protection from HIV through VMMC could lead clients to opt for risky sexual practices and reduce the ability for adopting options available for safer sex.

Further studies need to be done to ascertain if male circumcision has an impact on condom use and a study that incorporates pre and post circumcision sexual practices in participants.

## Data Availability

The data of this study are available without restriction from the corresponding author.

## Abbreviations

AIDS: Acquired Immune Deficiency Syndrome
ART: Antiretroviral therapy
CO: Commanding officer
COMREC: College of Medicine Research and Ethics Committee
DHO: District Health Office
HIV: Human Immunodeficiency Virus
IEC: Information, Education and Communication
JHPIEGO: John Hopkins Program for International Education in Gynecology and Obstetrics
MC: Male circumcision
MDHS: Malawi Demographic and Health Survey
MoH: Ministry of Health
PCI: Project Concern International
PI: Principal Investigator
RA: Research Assistant
STI: Sexually Transmitted Infections
UN: United Nations
UNAIDS: United Nations Programme on HIV and AIDS
VMMC: Voluntary Medical Male Circumcision
WHO: World Health Organization
